# Atlantoaxial subluxation as an early manifestation in an adolescent with undifferentiated spondyloarthritis: a case report and review of the literature

**DOI:** 10.1186/1752-1947-5-275

**Published:** 2011-07-03

**Authors:** Eyal Muscal, Krishna B Satyan, Andrew Jea

**Affiliations:** 1 Division of Pediatric Rheumatology, Texas Children's Hospital, Department of Pediatrics, Baylor College of Medicine, Houston, TX, USA; 2 Neuro-Spine Program, Division of Pediatric Neurosurgery, Texas Children's Hospital, Department of Neurosurgery, Baylor College of Medicine, Houston, TX, USA

## Abstract

**Introduction:**

Atlantoaxial instability has been described as a manifestation of ankylosing spondylitis (juvenile and adult onset), reactive arthritis, juvenile idiopathic arthritis, and rheumatoid arthritis; however, it has rarely been reported as an early manifestation of these disorders. We present this case report to increase awareness of the condition in the hope that earlier recognition of this disease may prevent further serious injury.

**Case presentation:**

We report the case of a 17-year-old Hispanic adolescent woman who was initially diagnosed with undifferentiated spondyloarthritis due to peripheral arthritis, enthesitis, a positive human leukocyte antigen B27 result, and inflammatory spinal pain lasting two months. Our patient experienced persistent and worsening occipitocervical pain and signs of myelopathy three months after diagnosis; consequently, we found atlantoaxial instability along with cervical spine bone erosion and pannus formation. She was treated surgically with a C1-2 posterior instrumented fusion and at six weeks post-operatively was started on tumor necrosis factor α blockade. Her occipitocervical symptoms subsided following surgery and initiation of immunomodulation.

**Conclusions:**

Our report serves to emphasize to pediatric and adult general practitioners, pediatricians, internists, family physicians, pediatric and adult rheumatologists and spine surgeons that atlantoaxial subluxation may be an early manifestation of spondyloarthritis, and that the condition is treatable by surgical intervention and immunomodulation.

## Introduction

Studies have reported that atlantoaxial instability can be a feature of juvenile and adult ankylosing spondylitis (AS) [1-4] reactive arthritis, juvenile idiopathic arthritis [5] and rheumatoid arthritis [6] in the course of the disease. Indeed, in two separate cohorts, 7% to 21% of adult patients with ankylosing spondylitis showed signs of subluxation as early as one year following AS diagnosis. However, there have been few previous descriptions of atlantoaxial subluxation as an early manifestation of undifferentiated spondyloarthritides (SpA) in adolescents.

Ankylosing spondylitis (AS), a form of adult spondyloarthritis, is a chronic rheumatic disorder characterized primarily by inflammation of the axial spine and sacroiliac joints and bone ossification. However, many individuals who also have features of peripheral arthritis and enthesitis (inflammation at tendon insertion sites) may not meet criteria for AS if their symptoms do not include axial involvement and radiographic evidence of sacroiliitis [7]; these individuals (adults and children less than 17 years of age) are often diagnosed with undifferentiated spondyloarthritis [8]. Features of childhood spondyloarthritis differ from those of adult onset disease and are characterized by more prevalent signs of peripheral arthritis affecting large joints (especially in the lower limbs) and enthesitis [9]. Indeed, children less than 17 years of age with features of undifferentiated spondyloarthritis may be diagnosed with enthesitis-related arthritis (ERA) according to the revised International League of Associations for Rheumatology (ILAR) classification system [10]. Atlantoaxial instability has been reported as a first sign in two cases of patients with juvenile ankylosing spondylitis (a seven-year-old human leukocyte antigen (HLA)-B27-negative girl [1] and an 11-year-old HLA-B27-positive boy), and two cases of patients with the previously utilized diagnosed schema for seronegative enthesitis and arthritis syndrome (SEA). We report a case of atlantoaxial subluxation as an early clinical manifestation of an undifferentiated form spondyloarthritis in a 17-year-old, HLA-B27-positive, adolescent girl.

## Case presentation

**Figure 1:**
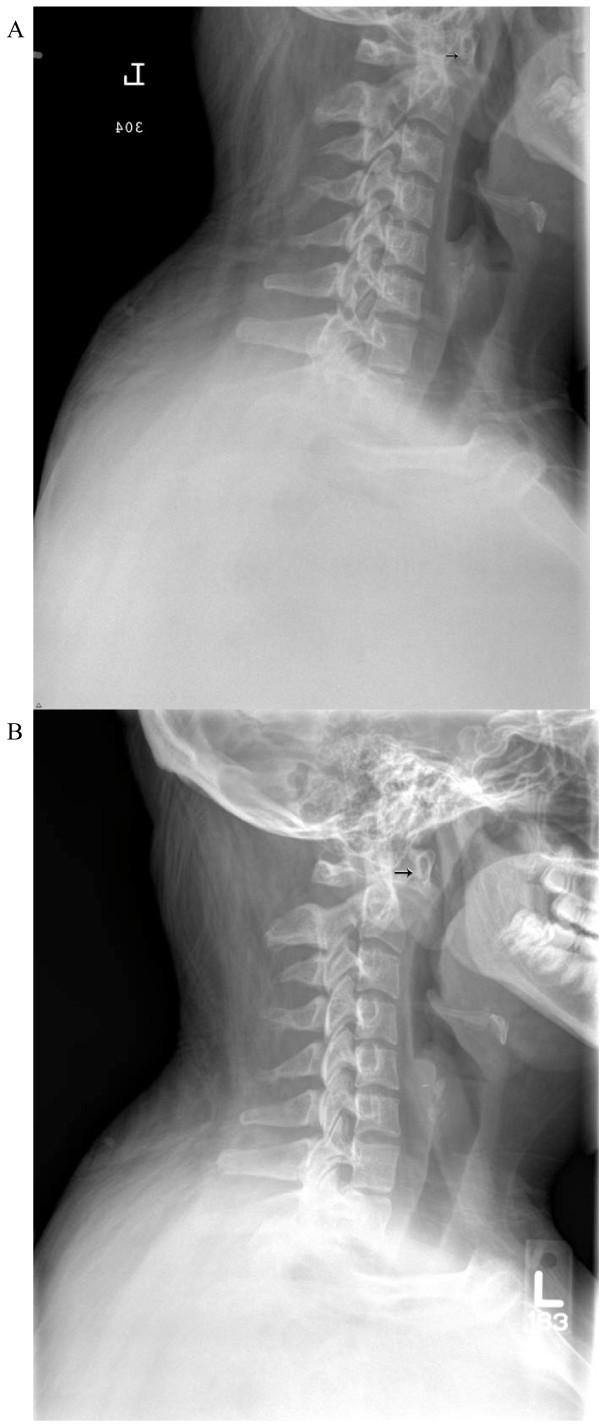
**A) Lateral cervical spine X-ray showing an atlantodental interval (arrow) of less than 6 mm**. B) Lateral cervical spine X-ray shows a progressive widening of the atlantodental interval (arrow) to more than 10 mm in the span of three months.

Our in-patient rheumatology service evaluated a 17-year-old Hispanic woman for pain in the neck, low back, bilateral hip, and knee as well as for headaches and morning stiffness lasting two months. At her initial hospitalization two weeks into the disease course, and at six weeks prior to rheumatology evaluation, the hospital service noted that she had decreased range of motion in her neck secondary to pain. Plain radiographic films of the cervical spine (Figure 1A) revealed no abnormalities, and she was discharged with a course of naproxen. Non-steroidal anti-inflammatory drugs (NSAIDs) offered only minimal relief of symptoms, which prompted an out-patient orthopedics community evaluation and resulted in diagnosis of 'gluteal strain' and bursitis. She was placed on propoxyphene/acetaminophen and cyclobenzaprine, but they did not improve her symptoms.

Due to the unremitting nature of our patient's symptoms two months into her illness, her pediatrician obtained laboratory studies and a bone scan which revealed abnormal uptake in the right seventh rib (possibly due to prior fracture), increased uptake in the left distal femur and anterior superior left tibial spine, and focal uptake at the facets of several thoracic vertebrae. Her erythrocyte sedimentation rate (ESR) was 49 mm/hour and high-sensitivity C reactive protein was 44.7 mg/L. Concerned about infection or malignancy, our patient's physician readmitted her to our institution. She did not have, then or previously, a history of fever, lymphadenopathy, bleeding or easy bruisability, weight loss, gastrointestinal symptoms, cardiac murmur, or rashes. Stool guaiac results were negative, and an abdominal ultrasound showed no abnormalities. A complete blood count was without cytopenias except for mild normocytic anemia.

**Figure 2:**
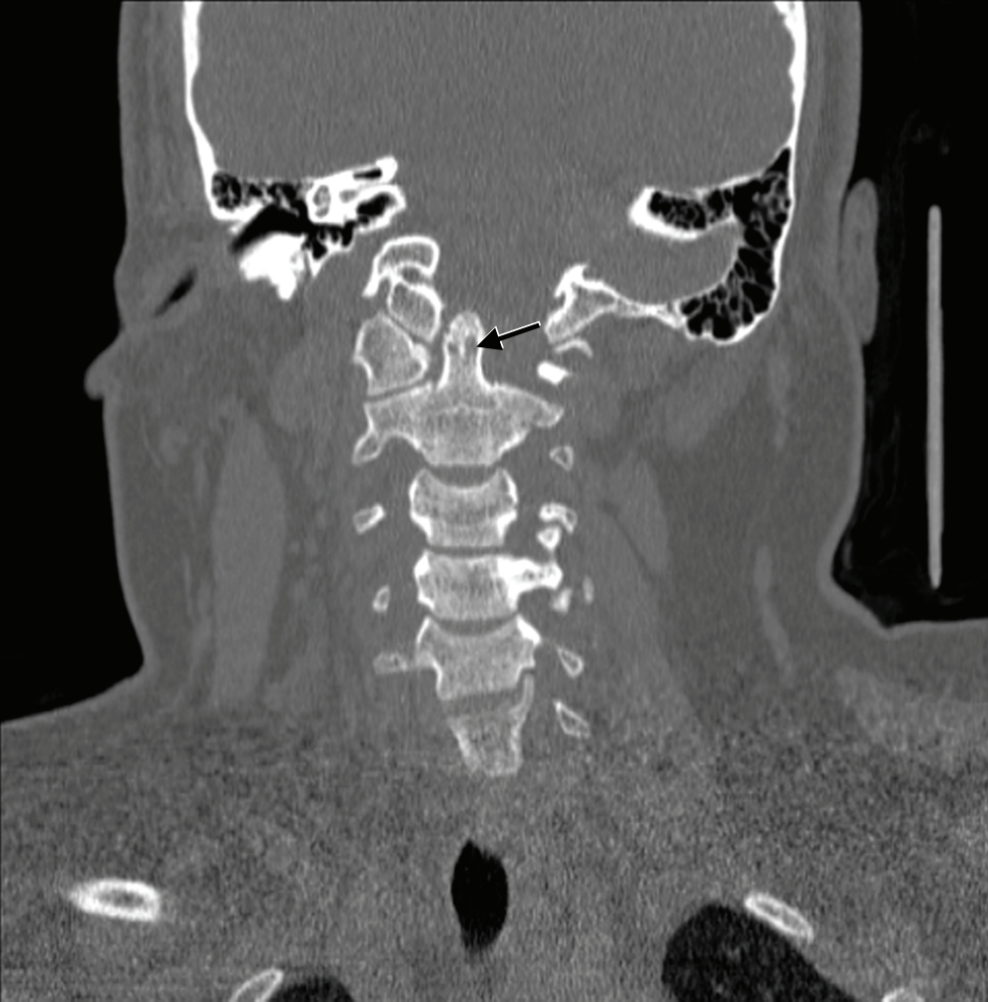
**Coronal computed tomography of the cervical spine shows evidence of bony erosion at the tip of the odontoid (arrow)**.

**Figure 3:**
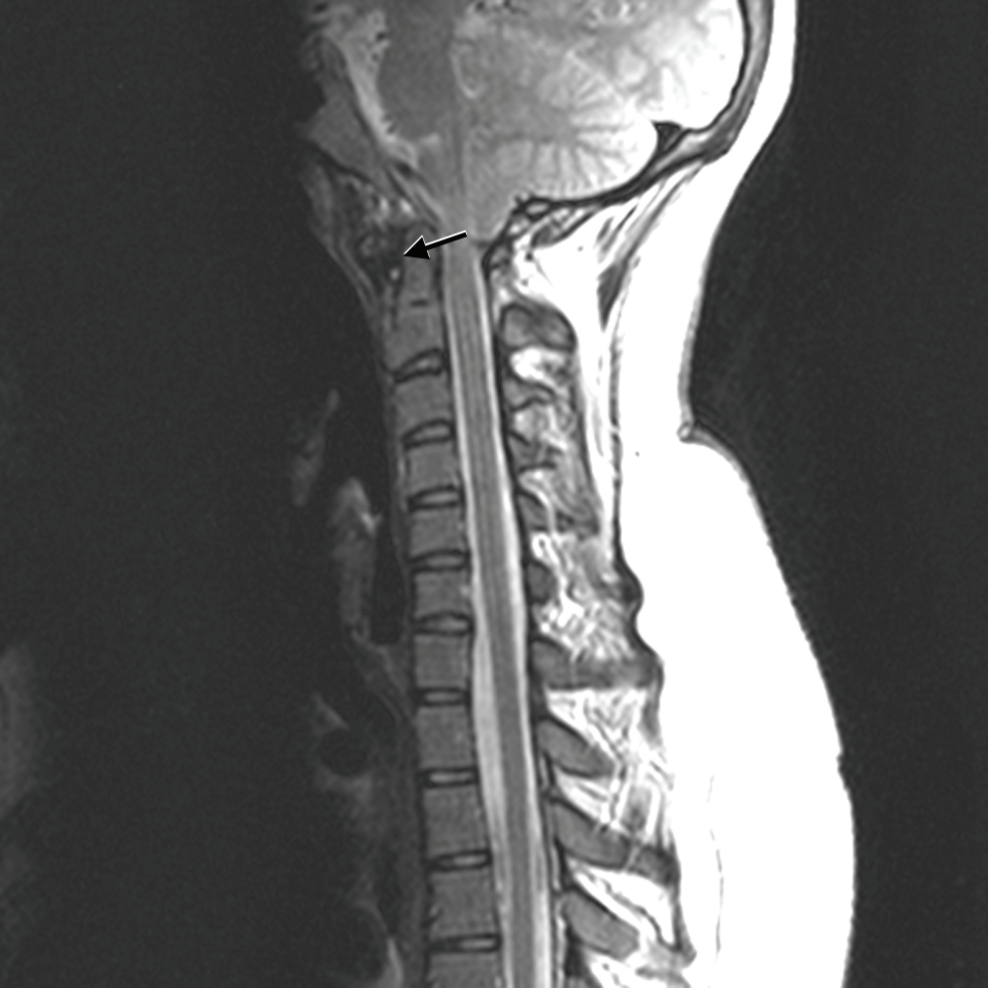
**Sagittal T2-weighted MRI of the cervical spine demonstrates widening of the atlantodental interval, cervical canal stenosis without spinal cord signal changes, and pannus formation (arrow)**.

A rheumatological evaluation revealed an obese (body mass index (BMI) 42, greater than 97%) adolescent with findings of point tenderness in her bilateral inferior patella, lower sacrum, and anterior hips. She had a reduced range of motion with muscle spasms in her neck, but no psoriatic lesions or nail pitting. Her spinal symptoms were most severe in the morning and improved with movement and NSAID use. Her family history was positive for idiopathic iritis (father) and inflammatory bowel disease with spondyloarthritis (paternal aunt). A rheumatoid factor was non-reactive, and anti-nuclear antibody was not detected. Creatine kinase and aldolase levels were within normal limits. A HLA-B27 marker was present in our patient. Imaging studies were consistent with an inflammatory process: a hip ultrasound revealed bilateral hip effusions, and a lower extremity MRI revealed T2 abnormal signals in patellar tendon insertions and subcutaneous tissue anterior to the inferior aspect of the left patellar tendon. Prior to her rheumatology evaluation, our patient was given celecoxib (as prescribed by her family physician), which provided significant pain relief in her back and reduction of morning stiffness. Our pediatric rheumatologist diagnosed our patient with undifferentiated spondyloarthritis using the European Spondyloarthropathy Study Group (ESSG) classification criteria (inflammatory spinal pain, hip synovitis, positive family history of HLA-B27-associated diseases, and enthesopathy/enthesitis). The family and our pediatric rheumatologists opted to keep our patient on celecoxib and have close out-patient follow-up because her musculoskeletal pain decreased and her inflammatory markers improved, Although her hip, back, and knee pain improved, our patient continued to have persistent neck pain with symptoms of occipital neuralgia after three months of scheduled NSAID therapy. New plain radiographic imaging of the cervical spine revealed a reversal of the normal lordotic curvature, and a 10 mm distance between the odontoid and anterior arches of C1 had markedly increased since prior films (Figure 1B). A computed tomography (CT) scan of the cervical spine showed evidence of bony erosion at the tip of the odontoid as well as mild rightward rotatory subluxation of C1, with moderate cervical stenosis at C1 and minimal flattening of the spinal cord (Figure 2). This was confirmed on MRI (Figure 3), which also demonstrated inflammation around the apical and transverse ligaments and adjacent pannus formation. There was no signal abnormality within the cord itself.

At this point, doctors were concerned about our patient's joint instability and referred her to the neurosurgery department. She had no recent history of travel, fever, pharyngitis, torticolis, or trauma. Results of a general examination showed our patient was obese but otherwise normal. She was awake and alert, with full strength throughout. Her left upper extremity was hyper-reflexive compared to her right upper extremity, and her right lower extremity was hyper-reflexive compared to her left lower extremity. Proprioception was intact. She had up-going toes bilaterally but no clonus or Hoffman sign. She had a steady gait with no sway on Romberg testing. Because clinical and radiographic evaluations showed evidence of atlantoaxial instability in the setting of undifferentiated spondyloarthritis, our neurosurgeons recommended a C1-2 fusion to our patient and her family. The doctors postulated that inflammation-mediated ligamentous laxity was causing joint instability but that ongoing infection did not cause the cervical spine disease (Grisel's syndrome). Her anti-inflammatory medication was stopped about one week prior to surgery.

### Surgical intervention

After our patient was fiber-optically intubated with in-line stabilization, we placed needle electrodes for intra-operative neurophysiological monitoring. Then, we measured and recorded baseline somatosensory-evoked potentials, motor-evoked potentials, and free-run electromyography (EMG) readings from the upper and lower extremities. Our patient was then positioned prone using the Mayfield three-point fixation system and a Jackson table; there was no change in her electrophysiology monitoring after positioning. Using fluoroscopy, we checked alignment of the cervical spine, finding a decrease in the atlantodental interval from pre-operative studies. Then, we made a midline incision over the spinous processes and dissected, in standard sub-periosteal fashion, the paraspinous muscle from the spinous processes and laminae. Subsequently, we isolated and bilaterally divided the C2 nerve roots and clearly identified bilaterally the C1 lateral masses, C2 pars, and C1-2 facet complexes. Under fluoroscopic guidance, we placed C1 lateral mass screws: a 4.0 × 34 mm screw on the right and 4.0 × 32 mm screw on the left (Vertex; Medtronic Sofamor Danek, Memphis, TN USA). Then, we placed bilateral, crossing, 3.5 × 24 mm translaminar C2 screws. We performed a C1 laminectomy to ensure that the cervical cord was well decompressed; decorticated the bone; and placed the C1 laminectomy autograft over the denuded surfaces using bone morphogenetic protein (Infuse; Medtronic Sofamor Danek, Memphis, TN USA) and bone matrix (Mastergraft; Medtronic Sofamor Danek) to supplement the graft. We then placed the rods, performed the final tightening, and closed the wound in a layered fashion. Intra-operative-evoked potentials revealed no changes during the case. There was no spontaneous EMG activity. In the immediate post-operative period after waking from general anesthesia, our patient was at her baseline examination levels.

### Post-operative course and immunomodulation

**Figure 4:**
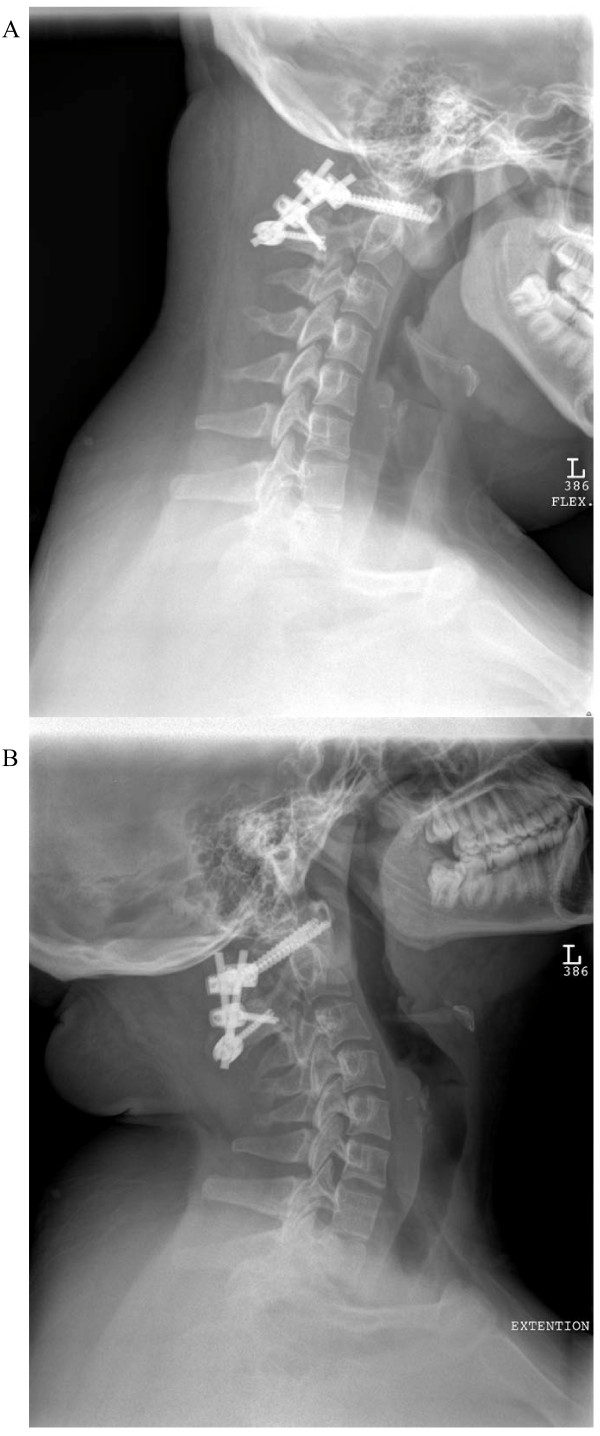
**A) Lateral cervical spine X-ray with flexion view at three months after surgery shows no abnormal movement between C1 and C2 vertebrae**. B) Lateral cervical spine X-ray with extension view at three months after surgery shows no abnormal movement between C1 and C2 vertebrae.

At two weeks after surgery, our patient was restarted on celecoxib. Poor wound healing and drainage required antibiotic coverage and delayed initiation of immunomodulation. At four weeks after surgery, our patient received a methylprednisolone infusion (1 g) and was started on adalimumab (40 mg subcutaneously every other week (actual text of hospital formulary)) eight weeks after her operation. She has had relief from neck pain and remains neurologically intact except for soft signs of myelopathy, which were found pre-operatively. There was no evidence of abnormal motion between the C1 and C2 vertebrae or evidence of instrumentation failure on dynamic cervical spine X-rays (Figure 4). While maintained on adalimumab, our patient has had intermittent complaints of hip and knee pain exacerbated by weather changes. Inflammatory markers have remained within normal limits since our patient started adalimumab (even during mild clinical flares). She has not developed psoriasis and there has been no evidence of sacroiliitis or irritable bowel disease (IBD) on MRI scans during a two-year follow up period.

## Discussion

### Nomenclature and classification schema

Spondyloarthritis in childhood and adolescence refers to a family of rheumatic diseases with overlapping clinical features that may cause peripheral arthritis and often enthesitis at an early age and may span through adulthood [8]. These conditions include undifferentiated spondyloarthritis, juvenile ankylosing spondylitis, psoriatic arthritis, reactive arthritis, and arthritis of inflammatory bowel disease. The estimated rates of both psoriatic arthritis and enthesitis-related arthritis (not including juvenile AS) are 0.28-0.88 cases per 100,000 children [11]. Based on adult AS prevalence data childhood-onset disease may be found in an additional 0.01% to 0.09% of children [11,12]. The spondyloarthritides are strongly associated with HLA-B27, an HLA class I major histocompatibility complex (MHC) gene that confers significant heritability in adult AS [8]. HLA-B27 is found in 90% of patients with AS but only 7% to 8% of healthy controls (only around 5% of HLA-B27 carriers develop AS).

### Juvenile ankylosing spondylitis

Juvenile ankylosing spondylitis (JAS) is clinically similar yet not identical to adult-onset disease. Juvenile and adolescent disease shows a higher frequency of extra-spinal joint involvement and enthesitis [9]. Diagnosis of JAS still requires radiographic evidence of sacroiliitis. Initial plain imaging findings of the sacroiliac regions and spine are often normal or difficult to interpret in children with spondyloarthritis manifestations, and the findings are mostly not sufficient to be labeled as ankylosing spondylitis according to the New York Criteria. These factors make it difficult to definitively diagnose ankylosing spondylitis in most children with features of spondyloarthritis.

### Undifferentiated spondyloarthritis

Pediatric spondyloarthritis diagnostic schemas underscore the higher prevalence of enthesitis in childhood disease (primary criteria) while downplaying less prominent rates of inflammatory back pain (primary criteria in ESSG and newer axial spondyloarthritis classification schema) [13-15]. Spine localization, enthesopathy, and lower limb involvement differentiate spondyloarthritis from other forms of juvenile idiopathic arthritis, excluding enthesitis-related arthritis (ERA). In such cases, as in our patient, the presence of family history, positive HLA-B27 result, peripheral arthritis, and enthesitis would support a spondyloarthritis diagnosis in both pediatric and adult classification schema.

According to ILAR pediatric juvenile idiopathic arthritis (JIA) criteria, patients with spondyloarthritis symptoms may be diagnosed with psoriatic arthritis (arthritis plus psoriasis or arthritis with two additional features: first degree relative with psoriasis, dactylitis, or nail pitting), ERA, or undifferentiated arthritis (combination of both psoriatic arthritis and ERA) [10]. ERA has an upper age limit of 16 but otherwise approximates adult spondyloarthritis schema. An ERA diagnosis requires a patient to have either (1) both arthritis and enthesitis or (2) either a physical finding and two additional historical or lab criteria. These criteria include: sacroiliac joint tenderness or inflammatory lumbosacral pain, HLA-B27 antigen, acute anterior uveitis, specific HLA-B27 associated disease family history, or arthritis in males over six years of age.

Adolescents over 16 years of age who have these clinical features may be diagnosed with spondyloarthritis using the similar ESSG schema, which require a patient to have either inflammatory spinal pain, or synovitis (asymmetrical or predominantly in lower limbs) as primary criteria plus one additional family history detail (including psoriasis or inflammatory bowel disease) or physical examination finding, which includes urethritis, alternating bilateral gluteal pain, enthesopathy, sacroilitis, cervicitis, or diarrhea a month prior to development of arthritis. Even the newer adult axial SpA Assessment of SpondyloArthritis International Society (ASAS) classification criteria for axial spondyloarthritis, which utilize MRI findings, rely on findings of back pain (more than three months duration in patients less than 45 years of age) as a primary clinical feature.

As in adult-onset disease, undifferentiated spondyloarthritis in childhood and adolescence may represent an early phase or incomplete form of ankylosing spondylitis or just be a related condition in the spectrum of immune-mediated arthritides that differ from rheumatoid arthritis. Patients with juvenile onset AS and back pain demonstrate radiographic evidence of sacroilitis within five to 10 years of disease onset [16]. This approximates the experience in adult spondyloarthritis cohorts in which fulfillment of AS criteria was observed in 25% to 36% of patients at five years [17,18]. More recent data suggest that many patients with undifferentiated spondyloarthritis may represent a distinct disease entity based on demographic and clinical criteria [19]. Indeed, one review suggests that progression from undifferentiated forms of spondyloarthritis to AS may only occur in 50% of juvenile and adult patients [20].

Efforts to diagnose pre-radiographical axial disease in adult patients concentrate on the differentiation of inflammatory back pain from mechanical etiologies by orthopedists, neurosurgeons, adult primary care physicians, and rheumatologists. A strict definition of inflammatory back pain, use of HLA-B27 and specific MRI findings are meant to allow for earlier diagnosis of axial spondyloarthritis and earlier use of immunomodulation (tumor necrosis factor (TNF) blockade) so to preclude the development of permanent structural damage [21]. Several studies have shown that TNF blockade improves clinical, biochemical, immunological, and MRI markers of inflammation in adults with undifferentiated spondyloarthritis [16]. In a randomized controlled trial of 26 patients with juvenile-onset SpA (ESSG criteria) the infliximab treated group had a significant reduction in median number of active joints at 12 weeks [22]. Due to the paucity of controlled clinical trials in the pediatric age group, current approaches to the treatment of spondyloarthritis in children and adolescents are often based on clinical experience and anecdotal findings. Guidelines for adult patients with AS, established by national and international research consortia, are currently only applicable to children who meet modified New York criteria of AS.

The clinical scenario of our patient resembles the characteristics of the undifferentiated spondyloarthritis group described above: she has not developed features of AS, psoriasis, or IBD, yet within the next five to 10 years she may develop typical radiographic changes of sacroilitis and bone ossification. To the best of our knowledge, only a few other children with similar presentations have been described in the literature [1,2,23].

### Atlantoaxial instability

This report highlights that atlantoaxial subluxation may be an early feature of spondyloarthritis in children and adolescents, even before the disease differentiates into juvenile ankylosing spondylitis. It is important that pediatricians, pediatric rheumatologists, family physicians, and pediatric spine surgeons recognize this clinical manifestation early to prevent serious neurological morbidity from spinal cord injury. Cervical spine X-rays and CT examination are essential. MRI may show proliferation of the synovium (pannus formation), myelomalacia, and cervical canal stenosis. Indications for surgical intervention include: symptoms and signs of cervical myelopathy, intractable mechanical occipitocervical neck pain, worsening of atlantoaxial instability (increased atlantodental interval ≥6 mm), previous MRI evidence of spinal cord injury (myelomalacia), and MRI evidence of persistent active inflammation despite immunomodulating medical therapy. Close follow-up is mandatory for young patients with spondyloarthritis in order to disclose other possible neurological or spinal manifestations such as spinal fracture, lumbar stenosis associated with neurogenic claudication or cauda equina syndrome, and progressive chin-on-chest deformity, which requires intervention at an early stage.

## Conclusions

Atlantoaxial instability may be an early manifestation of spondyloarthritis in children and adolescents. Symptomatic cases are treatable via surgical treatment by fusing of C1 and C2 and immunomodulation. It is important to closely follow-up with patients with spondyloarthritis to detect neurological or spinal complications of the disease that require aggressive medical management or neurosurgical intervention.

## Consent

Written informed consent was obtained from the patient's next-of-kin for publication of this case report and any accompanying images. A copy of the written consent is available for review by the Editor-in-Chief of this journal.

## Competing interests

The authors declare that they have no competing interests.

## Authors' contributions

EM and KBS were major contributors to the writing and editing of the manuscript. EM analyzed and interpreted the patient data regarding the rheumatic disease course. AJ was a major contributor in the design, writing, and editing of the manuscript. All authors read and approved the final manuscript.
